# 2505: Understanding quality of life transitions for women: Assessing the impact of EPIC decision support tools to address untreated menopausal symptoms on women’s quality of life and provider workflow

**DOI:** 10.1017/cts.2017.82

**Published:** 2018-05-10

**Authors:** Margaret Demment, Ivelisse Rivera, Morgan Pratte, Miriam Weber, Chris Morley, Tim Dye

**Affiliations:** University of Rochester Medical Center, Rochester, NY, USA

## Abstract

OBJECTIVES/SPECIFIC AIMS: The goal of this study is to assess how quality of life scores change in menopausal women before and after implementation of this aid. In addition, we are also interested in 2 process evaluation objectives: (1) determine if MyChart, the patient portal, is an effective way for this patient population to provide insight their quality of life to their providers and (2) to evaluate providers use of and reactions to the decision support tool. METHODS/STUDY POPULATION: This project is a collaboration between University of Rochester Medical Center and S.U.N.Y. Upstate Medical University. Participants were recruited through Upstate’s Family Medicine and OB/GYN practices via a MyChart invitation sent by the practices. Participating patients will be asked to complete a survey, through MyChart, every 3 months for 18 months. Participating health providers will be trained to use the decision support tool and participate in 3 interviews with the researchers to gain insight into the usefulness and effectiveness of the tool. RESULTS/ANTICIPATED RESULTS: Of the 465 eligible women, 117 women responded to our MyChart invitation to join our study. Of these, 105 agreed to participate and 98 met eligibility criteria. Only half of the women currently enrolled in our study had spoken to a provider about menopause related symptoms (56.1%) prior to study enrollment. DISCUSSION/SIGNIFICANCE OF IMPACT: The goal of this study is to improve menopause related symptoms in women, thus increasing their quality of life, but it will also provide important process evaluation for using EPIC and MyChart for future research studies.

